# MicroRNAs modulate immunological and inflammatory responses in Holstein cattle naturally infected with *Mycobacterium avium* subsp. *paratuberculosis*

**DOI:** 10.1038/s41598-023-50251-9

**Published:** 2024-01-02

**Authors:** Gerard Badia-Bringué, María Canive, Cristina Blanco-Vázquez, Rosana Torremocha, Susana Ovalle, Ricardo Ramos-Ruiz, Rosa Casais, Marta Alonso-Hearn

**Affiliations:** 1 Department of Animal Health, NEIKER-Basque Research and Technology Alliance (BRTA), Derio, Spain; 2 Doctoral Program in Molecular Biology and Biomedicine, Universidad del País Vasco/Euskal Herriko Unibertsitatea (UPV/EHU), Leioa, Bizkaia Spain; 3 Center of Animal Biotechnology, SERIDA-Regional Service of Agri-Food Research and Development, Deva, Spain; 4 Genomic Unit, Scientific Park of Madrid, Campus de Cantoblanco, Madrid, Spain

**Keywords:** Biological techniques, Gene expression analysis

## Abstract

MicroRNAs (miRNAs) regulate the post-transcriptional expression of genes by binding to their target mRNAs. In this study, whole miRNA sequencing was used to compare the expression of miRNAs in ileocecal valve (ICV) and peripheral blood (PB) samples of cows with focal or diffuse paratuberculosis (PTB)-associated lesions in gut tissues versus (vs) control cows without lesions. Among the eight miRNAs differentially expressed in the PB samples from cows with diffuse lesions vs controls, three (miR-19a, miR-144, miR32) were also down-regulated in cows with diffuse vs focal lesions. In the ICV samples, we identified a total of 4, 5, and 18 miRNAs differentially expressed in cows with focal lesions vs controls, diffuse lesions vs controls, and diffuse vs focal lesions, respectively. The differential expression of five microRNAs (miR-19a, miR-144, miR-2425-3p, miR-139, miR-101) was confirmed by RT-qPCR. Next, mRNA target prediction was performed for each differentially expressed miRNA. A functional analysis using the predicted gene targets revealed a significant enrichment of the RNA polymerase and MAPK signaling pathways in the comparison of cows with focal vs no lesions and with diffuse vs focal lesions, respectively. The identified miRNAs could be used for the development of novel diagnostic and therapeutical tools for PTB control.

## Introduction

Bovine paratuberculosis (PTB), often known as Johne’s disease, is a chronic granulomatous enteritis that affects ruminants worldwide. This disease is caused by *Mycobacterium avium* subsp. *paratuberculosis* (MAP) and is mainly characterized by a decrease in milk production and weight loss. Global economic losses from PTB cases are estimated to exceed US1.5 billion dollars per year^1^, with US198.42 million dollars in the United States and US364.31 million dollars in Europe^2^ primarily due to decreased milk production, increased management costs, and premature culling. Indeed, bovine PTB is considered endemic in the United States and Europe with more than 50% of herds being ELISA positive for anti-MAP antibodies^3^. In addition, scientific evidence links MAP to human inflammatory bowel disease (IBD), autoimmune diseases, as well as colorectal cancer and Alzheimer´s disease^4–6^. This potential threat to human health has stimulated interest in this disease and in the development of more sensitive diagnostic and control methods.

Transmission of MAP usually occurs early in the life of the animal by ingestion of MAP-contaminated feces or milk. MAP crosses the intestinal barrier via interaction with M and epithelial cells^7–9^ and can survive within subepithelial macrophages by inhibiting apoptosis and phagosome acidification, as well as by preventing antigens´ presentation to the immune system^10^. There are different stages of MAP infection (silent, subclinical, clinical, and advanced clinical) each with distinct immunological and pathological characteristics^11,12^. Th1 immune responses, in particular interferon-gamma (IFNɣ) production, are characteristics of silent infections, occur very early after infection, and can effectively contain MAP infections^13^. Around 10–15% of the infected animals eventually enter the subclinical phase of the infection where the Th1 responses gradually decline. This blockade of macrophage defenses allows MAP to survive and persist within infected host macrophages during the long-lasting subclinical stage of the infection. As the infection progresses, granulomas containing MAP have an impact on the intestinal mucosa and over time, they cause serious damage to the ileum and jejunum, diarrhea, progressive wasting, reduced milk yield, and the eventual death of the infected animal^14,15^. In the most advanced clinical stages of the infection, an ineffective Th2 humoral response arises and is indicative of MAP bacilli escaping granulomas and disseminating to various tissues and organs^16^. Current MAP control strategies generally rely on identifying and culling infected animals. The most common diagnostic tests are ELISA for the detection of anti-MAP antibodies and real-time PCR for the detection of MAP DNA in fecal samples. Although serum ELISA is a simple, fast, and a cost-effective method for diagnosis PTB, it is known to have low sensitivity for MAP-infected animals that do not show clinical signs^17^. The detection of subclinical infections remains a challenge and novel prognostic tools are needed to detect MAP-infected animals at the early stages of infection to control the spread of the disease.

RNA sequencing (RNA-Seq) is a high-throughput sequencing technique that provides data on the transcriptome of a cell or tissue. This technology has enormous potential because it enables the study of disease pathogenesis and can be used to identify biomarkers for the development of novel diagnostic tools, drugs, and vaccines^18^. RNA-Seq can provide biological information on how an animal is responding to an infection and be a source of novel biomarkers for early infection, disease progression, and therapy^19,20^. In the context of PTB, we previously identified host mRNAs differentially expressed in ileocecal valve (ICV) and peripheral blood (PB) samples of MAP-infected animals with distinct lesions in gut tissues^21^. The RNA that is not translated to protein is called non-coding RNA (ncRNA) and includes long-non coding RNAs (lncRNAs) and microRNAs (miRNAs), among others. LncRNAs are now emerging as important regulators of innate and adaptive immune responses, and there is growing evidence that lncRNAs contribute to the pathogenesis of human diseases like Crohn’s disease and inflammatory bowel disease^22–25^. MiRNAs are highly conserved small ncRNAs (18 to 25 nucleotides long) that regulate mRNA expression by binding to the 3’-untranslated region of their target mRNAs^26^. This binding results in either mRNA cleavage or protein translational repression. In contrast, miRNA binding to the 5’-untranslated regions (5’UTRs), exons, and even DNA elements may enhance translation and transcription^27^. Because miRNAs tend to associate with proteins and/or travel in serum and plasma within vesicles, extracellular miRNAs are more resistant to degradation than other RNAs or proteins and can, therefore, be measured with a high sensitivity^28^. The first studies showing miRNA expression in bovine tissues were conducted in 2007^29,30^. Since then, 793 mature miRNAs that are encoded on all 30 chromosomes, have been identified in the *Bos taurus* genome. These miRNAs make up about 25% of the 3825 ncRNAs predicted in the genome by Ensembl^31^. Previous studies found that certain miRNAs were differentially expressed in cattle infected with MAP *versus* (vs) uninfected cows, implying that certain miRNA profiles correlated with natural MAP infection^32^. Some of the previous studies used experimentally challenged cattle that might have a different miRNA profile compared with naturally infected animals^33–35^ and, therefore, the results of these studies must be validated in field studies and in animals naturally infected with MAP.

**Figure 1:**
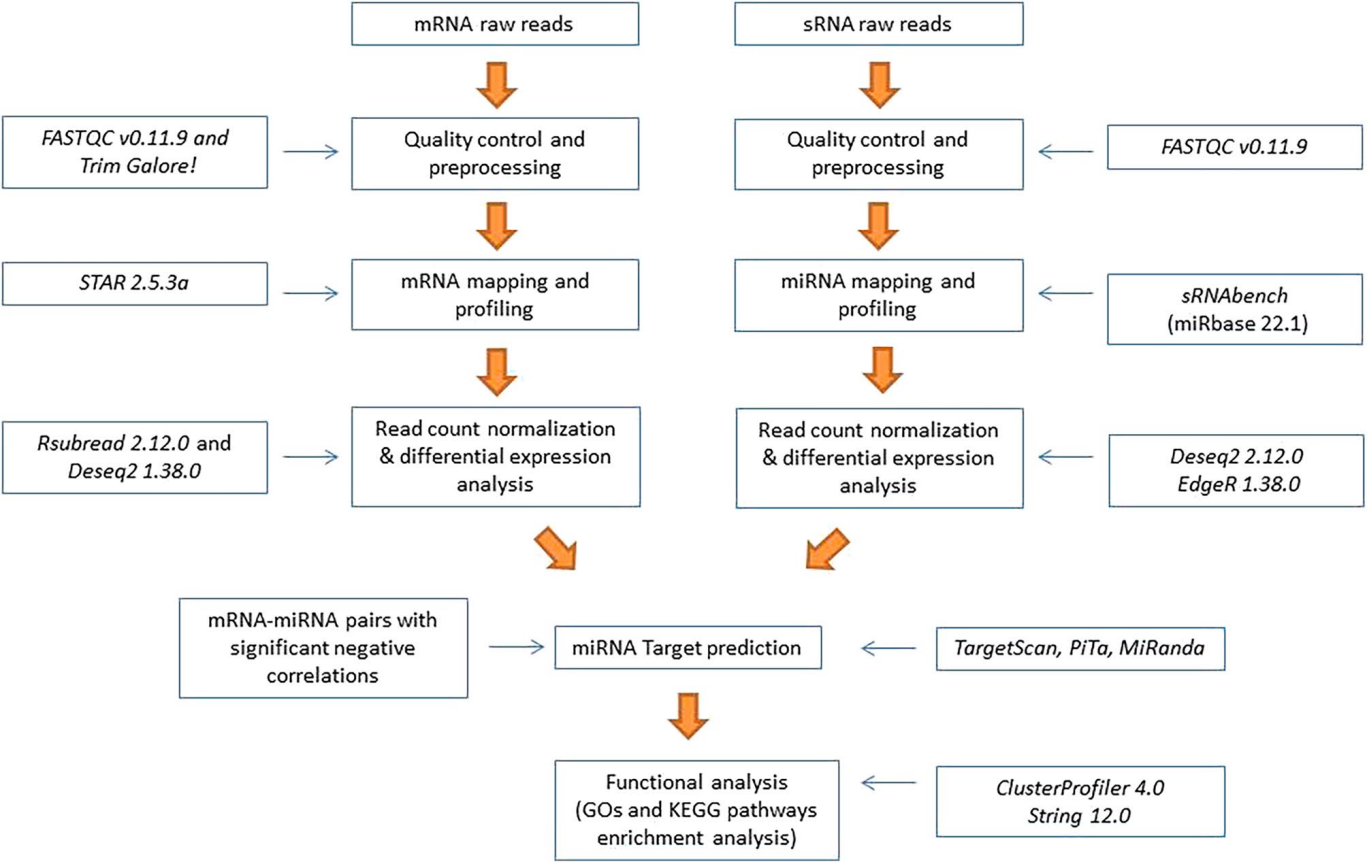
Bioinformatic pipeline. sRNA-Seq and RNA-Seq from PB and ICV samples of infected and control cows was performed, and the resulting reads were trimmed and aligned to the *Bos taurus* reference genome. Then, miRNA and mRNA differential expression was performed, and the results were integrated to identify a negative correlation between each differentially expressed miRNA and its predicted mRNA target(s).

In the current study, we used small RNA sequencing (sRNA-Seq) to sequence the small RNAs (sRNAs) of PB and ICV samples from 14 naturally infected Holstein cattle with distinct histopathological lesions in gut tissues. We compared the miRNA profiles of cows with focal and diffuse lesions vs cows without lesions and cows with focal vs diffuse lesions to identify differentially expressed miRNAs. We next performed a target prediction analysis using mRNA target databases and algorithms and built miRNA-mRNA pairs with significant negative paired correlations using mRNA expression data from the same animals. Using the predicted target genes, we performed a functional analysis to determine which pathways the differentially expressed miRNAs were involved in. The goal was to ascertain whether the differences in miRNA profiles would be reflected in the mRNA data set and explain differential disease outcomes. A schematic representation of the workflow is presented in Fig. 1.

## Materials and methods

### Ethical statement

All methods were carried out in accordance with relevant guidelines and regulations. The study is reported in accordance with ARRIVE guidelines (https://arriveguidelines.org). The Animal Ethics Committee of the Servicio Regional de Investigation y Desarrollo Agroalimentario (SERIDA) approved the procedures on the animals included in this study. All procedures were authorized by the Regional Consejería de Agroganadería y Recursos Autóctonos of the Principality of Asturias (approval code PROAE 29/2015 and PROAE 66/2019) and were carried out in accordance with the European Guidelines for the Care and Use of Animals for Research Purposes (2012/63/EU). PB, gut tissues, and fecal samples were collected by trained personnel and in accordance with good veterinary practices.

### Animals and PTB infectious status

The fourteen Holstein cows animals included in this study belonged to a single farm in Asturias (Spain). The infectious status of these animals was determined by histopathological analysis of gut tissues, ELISA for the detection of anti-MAP antibodies, and fecal and gut tissues PCR and bacteriological culture as previously described^21,36^. According to their location and extension, inflammatory cell type, and mycobacterial load, PTB-associated histopathological lesions were classified in focal and diffuse as previously described^14^. The average age of the animals without lesions and with focal and diffuse lesions was 5.45, 5.09, and 4.38 years old, respectively.

### RNA extraction, RNA-Seq library preparation, sequencing, and differential expression analysis

Total RNA isolation, RNA-Seq library preparation, and sequencing were previously performed^21^. The raw reads were filtered by length (> 75 bp) and by the percentage of ambiguous bases (< 10%) using *FastQC v0.11.9*^37^ and had PCR primers and Illumina adapters trimmed with *Trim Galore*^38^. The fastq reads (NCBI Gene Expression Omnibus database; Acc. Number GSE137395) were mapped against the most recent *Bos taurus* reference genome sequence (ARS-UCD1.2) using the Spliced Transcripts Alignment to a Reference aligner (*STAR 2.5.3a*)^39^. The alignments files (.bam) from *STAR* were used to generate a table of counts for each gene using the *FeatureCounts* function from *Rsubread 2.12.0*^40^. Gene counts were then normalized with the mean-of-ratios method of *DESeq2 1.38.0*^41^. In the current study, *DESeq2* was also used for the differential gene expression analysis. An mRNA was differentially expressed if its false discovery rate (FDR)-adjusted *p*-value was lower than 0.05.

### sRNA extraction, sRNA-Seq library preparation, and sequencing.

MiRNAs were isolated from PB samples using the PAXGene miRNA isolation kit following the instructions of the manufacturer (Qiagen). Total RNA from gut tissue samples was extracted with the miRNeasy Mini Kit according to the manufacturer’s protocol (Qiagen). For each sample, 150 ng of total RNA was used to prepare a sRNA-Seq library using the NEB-Next Small RNA Sample Preparation following the manufacturer´s protocol (New England Biolabs, Ipswich, MA, US). Each of the generating libraries was run through a mini-PROTEAN TBE Precast gel 5% (BioRAD, Madrid, Spain), and fragments from 140 to 325 bp of the library were excised from the gel and purified. A unique pool containing all the purified libraries was prepared and sequenced in 101 bp single-read in a NovaSeq 6000 sequencer (Illumina) at the Genomic Unit of the Scientific Park of Madrid, Spain.

### miRNAs differential expression analysis

Quality control of the raw reads was performed using *FastQC v0.11.9*^37^. Trimmed reads were analyzed using *sRNAtoolbox*, which consists of independent tools for sRNA analysis^42,43^. *sRNAbench,* a tool from *sRNAtoolbox,* was used to filter and align the trimmed reads to the *Bos taurus* genome (ARS-UCD1.2). Reads with less than 15 bp and with a mean phred score below 20 were filtered out of the analysis, and the maximum number of allowed mismatches in the mapping process was 1. *miRbase 22.1* database was used to annotate the miRNAs, and other ncRNAs were annotated using *RNAcentral 20.0* and *Ensembl* release 104 databases. For the normalization and differential expression analysis of the miRNAs, the alignments obtained from *sRNAbench* were fed directly to *sRNAde*, another web-based tool from *sRNAtoolbox*^42^. *sRNAde* uses different algorithms (*DESeq2 2.12* and *EdgeR 1.38.0*) to test for differential expression. A miRNA was differentially expressed if its FDR-adjusted *p*-value based on Benjamini and Hochberg testing correction^44^ was lower than 0.05 in the comparisons: with focal lesions vs controls, with diffuse lesions vs controls, or with diffuse vs focal lesions.

### Reverse transcription quantitative PCR (RT-qPCR) for RNA-Seq validation

Changes in the expression of five of the differentially expressed miRNAs were validated by RT-qPCR. For specific and highly sensitive RT-qPCR detection of a mature miRNA, 10 ng of total RNA was reverse transcribed into cDNA using the miRCURY LNA RT kit (Qiagen) according to the manufacturer´s instructions. For the RT reactions, a total volume of 10 µl (2 × MiRCURY RT Enzyme mix, reaction buffer, target RNA, and RNase-free water) was incubated for 60 min at 42 °C to perform the RT and 5 min at 95 °C to inactivate the reverse transcriptase. RT control reactions without the enzyme mix were included. The resultant cDNAs (1:40 dilution) were amplified on a LightCycler^®^ 480 thermal cycler using the miRCURY SYBR^®^ Green PCR Kit (Qiagen) in the presence of miRNA-specific and locked nucleic acid (LNA)-enhanced forward and reverse primers (Qiagen). PCR conditions were: 95 °C for 2 min (heat activation), followed by 40 cycles of 95 °C for 5 s (denaturation) and 56 °C for 30 s (annealing and extension), and a final melting curve. Appropriate controls (no template) were included. RT-qPCR experiments were performed by duplicate and in each experiment three replicates were run for each combination of primers and samples.

To obtain Ct values, the 2nd derivative max method of the LightCycler^®^ 480 Software was used. Using the results of the samples from cows without lesions or with focal lesions as controls, fold changes in expression were calculated using the 2^–ΔΔCt^ method. Normalization was performed using the U6 snRNA and 5S rRNA endogenous reference genes. Two comparisons were made: samples of cows with diffuse lesions vs controls and with diffuse vs focal lesions. The results were expressed as fold changes and were standardized by log_2_ transformation to be comparable to the sRNA-Seq differential expression results. To test if the differences between the groups were statistically significant, an unpaired t-test was run using *R*^45^. The Pearson correlation coefficient between the sRNA-Seq and RT-qPCR quantitative results was calculated using *R*. Differences and correlations were considered statistically significant if the *p*-value was ≤ 0.05.

### miRNA-gene target prediction

The putative target mRNAs for each miRNA were predicted using the tool *mRNAconsTarget* from *sRNAtoolbox* with the *miRanda*^46^ and *PITA*^47^ algorithms with default parameters. In parallel, the prediction database *TargetScan*^48,49^ was used to predict mRNA targets with a score ≥ 0.2. To reduce potential false positive targets, only targets predicted by the three algorithms were considered for further analysis. Next, the correlations between the miRNAs and predicted mRNA expression levels were tested with the Spearman and Pearson correlation tests using *R*^45^. Using both correlation tests, mRNAs with a negative correlation (R < 0 and *p-*value ≤ 0.05) with respect to their related miRNA were considered as putatively affected by the miRNA expression.

### Functional analysis

Using the predicted gene targets for each comparison, an enrichment analysis of gene ontology (GO) processes and Kyoto Encyclopedia of Genes and Genomes (KEGG) pathways was performed using *ClusterProfiler* 4.0^50^ and *String 12.0*^51^. An FDR-adjusted *p*-value lower than 0.05 was considered significant. The function of the candidate genes was searched in *GeneCards* by searching their gene symbol.

## Results

### sRNA-seq data

The animals included in the study were tested by histopathological analysis of gut tissues, acid-fast stain (Zielh-Neelsen), specific antibody response against MAP measured by ELISA, fecal and tissue qPCR, and bacteriological culture as previously described ^21,36^. The infectious status of the 14 animals included in the study are presented in Table 1. The four control animals had no histopathological lesions in gut tissues and had a negative result for ELISA, fecal and tissue bacteriological culture, and qPCR. All the infected animals had focal or diffuse lesions in gut tissues. The five animals with focal lesions in gut tissues had a positive tissue qPCR, none of them had heavy bacterial load (> 50 cfu/g) in feces and tissues, and none of them was culled due to PTB-associated clinical signs. Only one of the animals with focal lesions was fecal PCR positive. All the animals with diffuse lesions had positive ZN and ELISA results, positive fecal and tissue qPCR results, and positive bacteriological culture from gut tissues. Four of the five animals with diffuse lesions had heavy bacterial load in gut tissues.

**Table 1:** Results of the histopathological analysis, ZN stain, ELISA, PCR, and bacteriological culture from the 14 animals included in the study.

Animal ID	Histopathological analysis			ELISA	Fecal PCR	Fecal culture	Tissue PCR	Tissue culture
	Microscopic	Macroscopic	ZN					
1	Negative	No	Negative	Negative	Negative	Negative	Negative	Negative
2	Negative	No	Negative	Negative	Negative	Negative	Negative	Negative
3	Negative	No	Negative	Negative	Negative	Negative	Negative	Negative
15	Negative	No	Negative	Negative	Negative	Negative	Negative	Negative
4	Focal	No	Negative	Negative	Negative	Negative	Positive	Negative
5	Focal	No	Negative	Negative	Negative	Negative	Positive	Low
6	Focal	No	Negative	Negative	Negative	Negative	Positive	Negative
7	Focal	No	Negative	Negative	Negative	Negative	Positive	Negative
8	Focal	No	Negative	Positive	Positive	Negative	Positive	Medium
10	Diffuse	Yes	Positive	Positive	Positive	Negative	Positive	Heavy
11	Diffuse	Yes	Positive	Positive	Positive	Negative	Positive	Heavy
12	Diffuse	Yes	Positive	Positive	Positive	Heavy	Positive	Heavy
13	Diffuse	Yes	Positive	Positive	Positive	Negative	Positive	Low
14	Diffuse	Yes	Positive	Positive	Positive	Negative	Positive	Heavy

Bacterial load was classified as low (< 10 CFU), medium (between 10 and 50 CFU) or heavy (> 50 CFU).

*N* Ziehl–Neelsen, *CFU* colony forming units.

**Figure 2:**
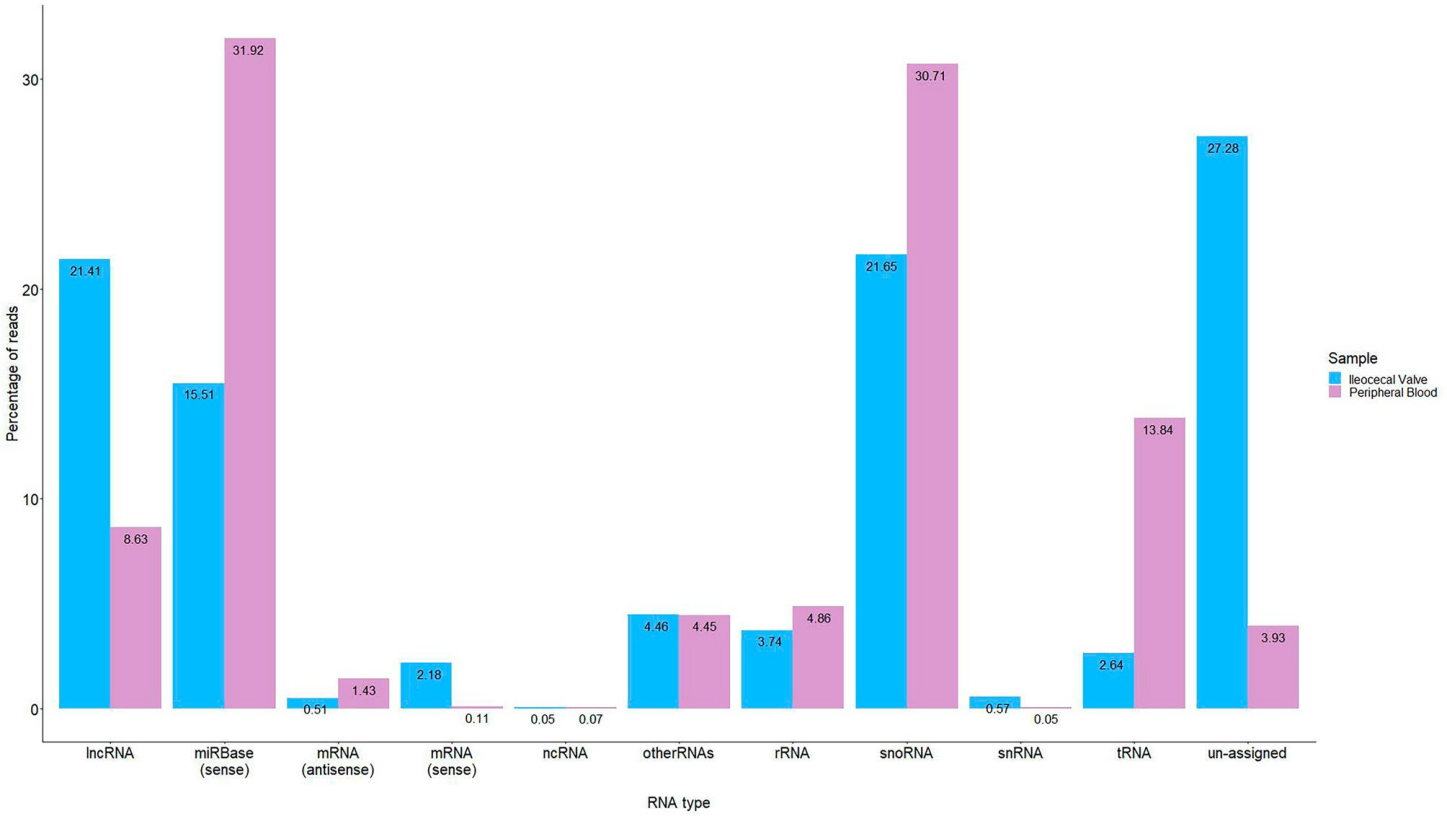
Categories of the identified ncRNAs in peripheral blood and ileocecal valve samples. The reads that were not identified as miRNAs were long-non coding RNAs (lncRNAs), transfer RNAs (tRNAs), small nuclear RNAs (snRNAs), small nucleolar RNAs (snoRNAs), ribosomal rRNA (rRNA), and other and unassigned RNAs.

sRNA-Seq libraries were prepared from total RNA extracted from PB and ICV samples of the 14 animals which were classified into three groups according to the results of the histopathological analysis: animals with focal and diffuse lesions and animals without lesions in gut tissues and regional lymph nodes (controls). An average of 40,954,530 and 36,762,469 raw reads per library were obtained after sequencing the PB and ICV libraries, respectively, and an average of 35.8 million reads per library (92.4%) passed the quality control process. The alignment of the filtered reads resulted in 34.2 million mapped reads (94.6%) per library. Mapped reads were annotated using miRBase v22 to identify known miRNAs in PB and ICV samples, and 31.92 and 15.51 million reads mapped to known miRNAs in the miRBase v22, respectively. The remaining reads that were not identified as miRNAs were lncRNAs, transfer RNAs (tRNAs), small nuclear RNAs (snRNAs), small nucleolar RNAs (snoRNAs), ribosomal rRNA (rRNA), and unassigned RNAs (Fig. 2).

### miRNA differential expression analysis

A total of 420 and 358 mature miRNAs identified in the PB and ICV samples were analysed for differential gene expression with the *EdgeR* and *DESeq2* algorithms. In general, there were more miRNAs with decreased than increased expression levels in all the comparisons (Tables 2 and 3). As expected, the number of differentially expressed miRNAs was larger in the ICV than in the PB samples. None of the miRNAs was differentially expressed in both the PB and ICV samples and, therefore, our results showed PB and ICV-specific miRNA expression. A list of the differentially expressed miRNAs identified with the *EdgeR* and/or *DESeq2* algorithms in the PB samples of the infected animals is presented in Table 2. In the comparison of focal vs controls, we did not identify any dysregulated miRNA. In the PB samples from animals with diffuse lesions vs controls, the consensus of the two algorithms revealed five miRNAs; two upregulated (bta-miR-2425-3p and bta-miR-139) and three downregulated (bta-miR-27a-5p, bta-miR-144, and bta-miR-181a). In this comparison, *DESeq2* was able to identify an additional bta-miR-32 (fold = −2.62) and the *Edge2* identified the bta-miR-27a-5p (fold = −3.68) and bta-miR-181a (fold = −1.86). The bta-miR-2425-3p was the most upregulated miRNA in our study with a fold of 5.76. Among the eight miRNAs differentially expressed in PB samples from cows with diffuse lesions vs controls, three downregulated miRNAs (bta-miR-19a, bta-miR-144, bta-miR-32) were also found downregulated in PB samples of cows with diffuse vs focal lesions.

**Table 2:** List of the differentially expressed miRNAs identified with the *EdgeR* and *DESeq2* algorithms in the peripheral blood samples of animals with diffuse lesions vs controls and animals with diffuse vs focal lesions.

Comparison	Algorithm	miRNA name	Fold change (log_2_)	Adjusted *p*-value
Diffuse lesions vs controls	*DESeq2*	bta-miR-2425-3p	5.760949308	0.000901
		bta-miR-144	−3.614670202	0.002987
		bta-miR-19a	−2.643658463	0.011826
		bta-miR-32	−2.628257174	0.049391
		bta-miR-139	1.841533295	0.049391
		bta-miR-101	−2.375232972	0.049391
	*EdgeR*	bta-miR-144	−3.523181922	0.000791
		bta-miR-27a-5p	−3.686888682	0.003369
		bta-miR-2425-3p	5.698593367	0.005617
		bta-miR-19a	−2.5239869	0.011452
		bta-miR-101	−2.218090784	0.036952
		bta-miR-139	1.914828963	0.036952
		bta-miR-181a	−1.860477015	0.036952
Diffuse vs focal lesions	*DESeq2*	bta-miR-19a	−3.071981051	2.96E−05
		bta-miR-144	−3.798502613	2.96E−05
		bta-miR-32	−2.958950883	0.001641
	*EdgeR*	bta-miR-144	−3.91750149	0.001931
		bta-miR-19a	−3.182992869	0.001931
		bta-miR-32	−3.05395378	0.003893

**Table 3:** List of the differentially expressed miRNAs identified with the *EdgeR* and *DESeq2* algorithms in ileocecal valve samples of infected animals with focal or diffuse lesions vs controls and with diffuse vs focal lesions.

Comparison	Algorithm	miRNA	Fold change (log_2_)	Adjusted *p*-value
Focal lesions vs controls	*DESeq2*	bta-miR-150	1.72663	0.03206
		bta-miR-23b-3p	−1.11804	0.04662
		bta-miR-2478	3.23118	0.04662
		bta-miR-23a	−0.92494	0.04662
Diffuse lesions vs controls	*DESeq2*	bta-miR-146a	1.21000	0.00001
		bta-miR-433	−2.81867	0.01386
		bta-miR-99a-5p	−0.80058	0.01608
		bta-miR-135b	−4.45335	0.01608
	*EdgeR*	bta-miR-135b	−5.50608	0.01547
		bta-miR-215	2.60383	0.01547
		bta-miR-433	−2.76018	0.01547
Diffuse vs focal lesions	*DESeq2*	bta-miR-204	−2.38038	0.00173
		bta-miR-146b	2.16842	0.00917
		bta-miR-146a	1.91842	0.00969
		bta-miR-99a-5p	−1.68395	0.00969
		bta-miR-10174-3p	−1.83497	0.00969
		bta-miR-154c	−1.29954	0.00969
		bta-miR-433	−3.24259	0.00969
		bta-miR-23b-3p	−1.19803	0.01058
		bta-let-7e	−1.59002	0.01184
		bta-miR-214	−0.89678	0.02824
		bta-miR-23a	−0.88296	0.03594
		bta-miR-382	−1.66611	0.04663
		bta-miR-145	−1.92401	0.04663
		bta-let-7c	−1.73719	0.04663
		bta-miR-30a-5p	−1.21416	0.04663
		bta-miR-27b	−0.80365	0.04663
		bta-miR-10167-3p	2.54170	0.04663
		bta-miR-411a	−1.02399	0.04663

The list of the differentially expressed miRNAs identified in the ICV samples with the *EdgeR* and *DESeq2* algorithms is presented in Table 3. The bta-miR-2478 (fold = 3.23) was the most highly upregulated miRNA in the tested ICV samples. In the ICV samples, we identified a total of 4, 5, and 18 miRNAs differentially expressed in cows with focal lesions vs controls, diffuse lesions vs controls, and diffuse vs focal lesions, respectively. In the comparison of focal lesions vs controls, *DESeq2* was able to identify two upregulated (bta-miR-2478 and bta-miR-150) and two downregulated (bta-miR-23a and bta-miR-23b-3p) miRNAs. In the ICV samples of cows with diffuse lesions, both *DESeq2* and *EdgeR* identified two downregulated miRNAs (bta-miR-135b and bta-miR-433) when compared with control cows. In this comparison, *DESeq2* was able to identify two additional downregulated miRNAs (bta-miR-146a and bta-miR-99a-5p) and *EdgeR* identified the bta-miR-215 (fold = 2.60). Some of the miRNAs that were differentially expressed in this comparison (bta-miR-433, bta-miR-146a, bta-miR-99a-5p) were also dysregulated in the comparison of diffuse vs focal lesions. Two miRNAs (bta-miR-23a and bta-miR-23b-3p) were downregulated in the comparisons; focal lesions vs controls and diffuse vs focal lesions.

### Validation of the differentially expressed miRNAs in PB samples by RT-qPCR

**Figure 3:**
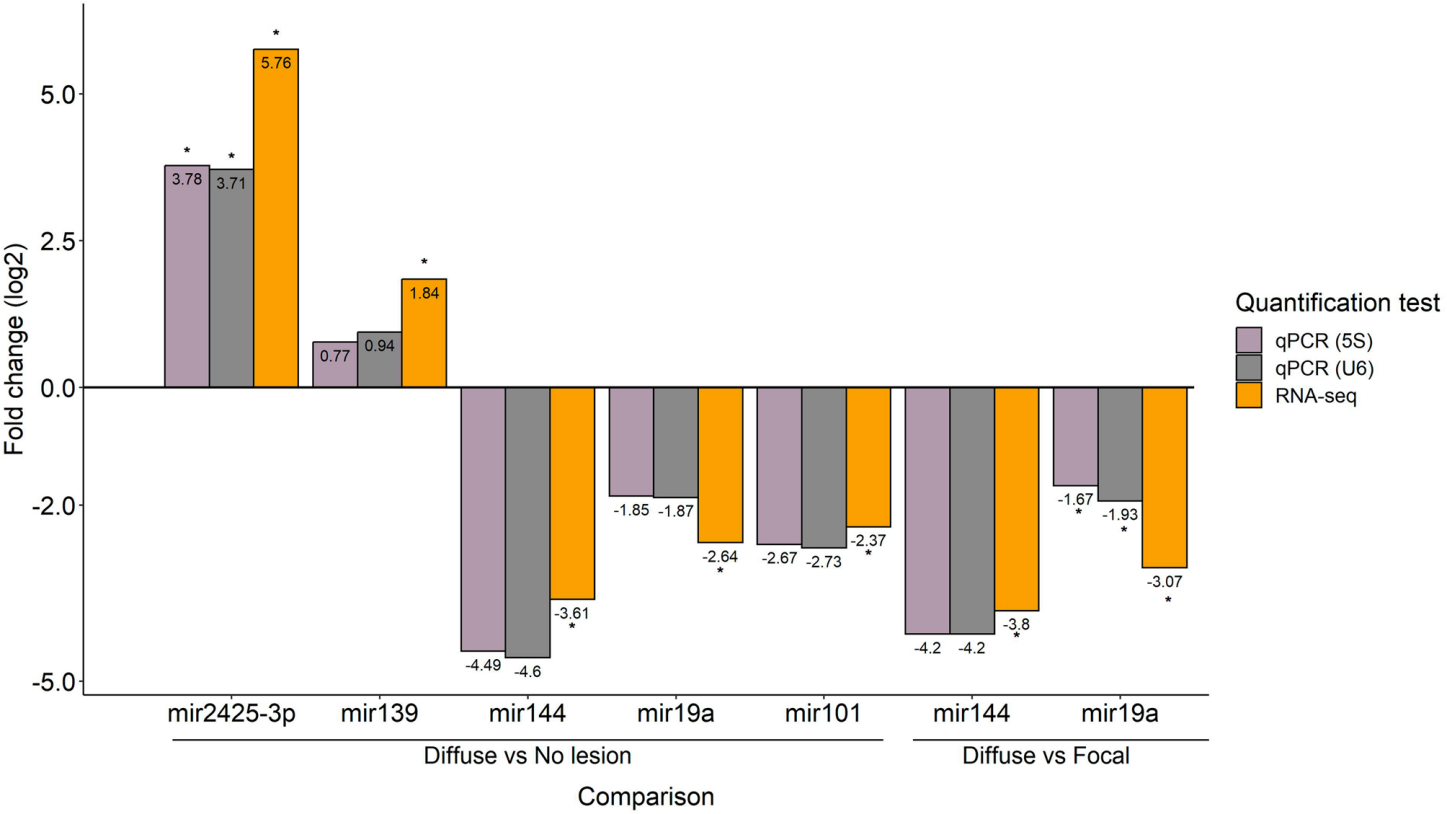
Validation of the differentially expressed miRNAs in PB samples by RT-qPCR. Validation of the differences in expression of five miRNAs (bta-miR-101-3p, bta-miR-19a-3p, bta-miR-139, bta-miR-144, bta-miR-2425-3p*)* was performed by RT-qPCR**.** Changes in the expression levels (expressed as the log_2_ of the fold change) of the selected differentially expressed miRNAs in the comparisons diffuse vs controls and with diffuse vs focal lesions was calculated using RNA-Seq (in yellow) and RT-qPCR. RT-qPCR results using the U6 snRNA or 5S rRNA as endogenous genes are presented in grey and purple, respectively. Significant changes in the miRNA expression (*p-*value ≤ 0.05) for each comparison are indicated with an asterisk.

The expression changes observed in five of the differentially expressed microRNAs (miR-19a, miR-144, miR-2425-3p, miR-139, miR-101) were validated by RT-qPCR using total RNA extracted from PB samples of the animals included in the study (Fig. 3). The selected miRNAs were all identified with both *DESeq2* and *EdgeR* algorithms. The bta-miR-2425-3p and bta-miR-139 were selected because they were the only miRNAs significantly upregulated in PB samples. The bta-miR-19a and bta-miR-144 were selected because they were found downregulated in all the tested comparisons. The results of the qRT-PCR are presented in Fig. 3 and were expressed as fold changes and standardized by log_2_ transformation to be comparable to the miRNA-Seq differential expression results analyzed with *DESeq2*. Using multiple reference genes for normalization minimizes technical variation^52^. Therefore, the normalization of the RT-qPCR results was performed using the expression of the U6 snRNA and 5S rRNA as the endogenous reference genes. As seen in Fig. 3 similar results were obtained when the U6 snRNA or 5S rRNA were used as endogenous genes for data normalization. The RT-qPCR results showed a statistically significant upregulation of the bta-miR-2425-3p in the comparison of cows with diffuse lesions vs controls (*p*-value ≤ 0.05). In the comparison of diffuse vs focal lesions, a statistically significant downregulation of the bta-miR-19a was observed (*p*-value ≤ 0.05). A similar trend (upregulation or downregulation) was observed in the results obtained with both RNA-Seq and RT-qPCR methods. Indeed, the Pearson correlation coefficient calculated for the miRNA-Seq and RT-qPCR data was equal to 0.978 and 0.959 when the U6 snRNA or 5S rRNA were used as endogenous genes; *p*-values = 0.003 and 0.0006, respectively.

### miRNA-gene target prediction

Potential target mRNAs of the differentially expressed miRNAs were predicted using three different algorithms (*miRanda, PiTA, TargetScan*). All the consensus mRNA targets were then investigated for negative Spearman and Pearson correlations with corresponding miRNA using mRNA sequencing data from the same animals. The number of differentially expressed miRNAs and their predicted mRNA targets for each comparison is presented in Table 4. In the PB samples, 35 and 32 mRNA targets were identified in the comparisons: diffuse lesions vs controls and diffuse vs focal lesions, respectively. In ICV samples, 33, 2, and 198 mRNA targets were identified in the comparisons focal lesions vs controls, diffuse lesions vs controls, and diffuse vs focal lesions, respectively.

**Table 4:** Number (No.) of differentially expressed (DE) miRNAs in peripheral blood (PB) and ileocecal valve (ICV) for each comparison and their predicted mRNA targets identified with *PiTA*, *miRanda*, and *TargetScan* algorithms and with a negative Spearman and Pearson correlation.

Sample	Comparison	No. of DE miRNAs	No. of DE miRNAs with targets	No. of targets (Pearson correlation)	No. of targets (Spearman correlation)	No. of total targets
PB	Focal lesions vs controls	0	0	0	0	0
	Diffuse lesions vs controls	8	4	11	32	35
	Diffuse vs focal lesions	3	2	10	30	32
ICV	Focal lesión vs controls	4	4	28	19	33
	Diffuse lesions vs controls	5	1	0	2	2
	Diffuse vs focal lesions	18	16	152	171	198

**Figure 4:**
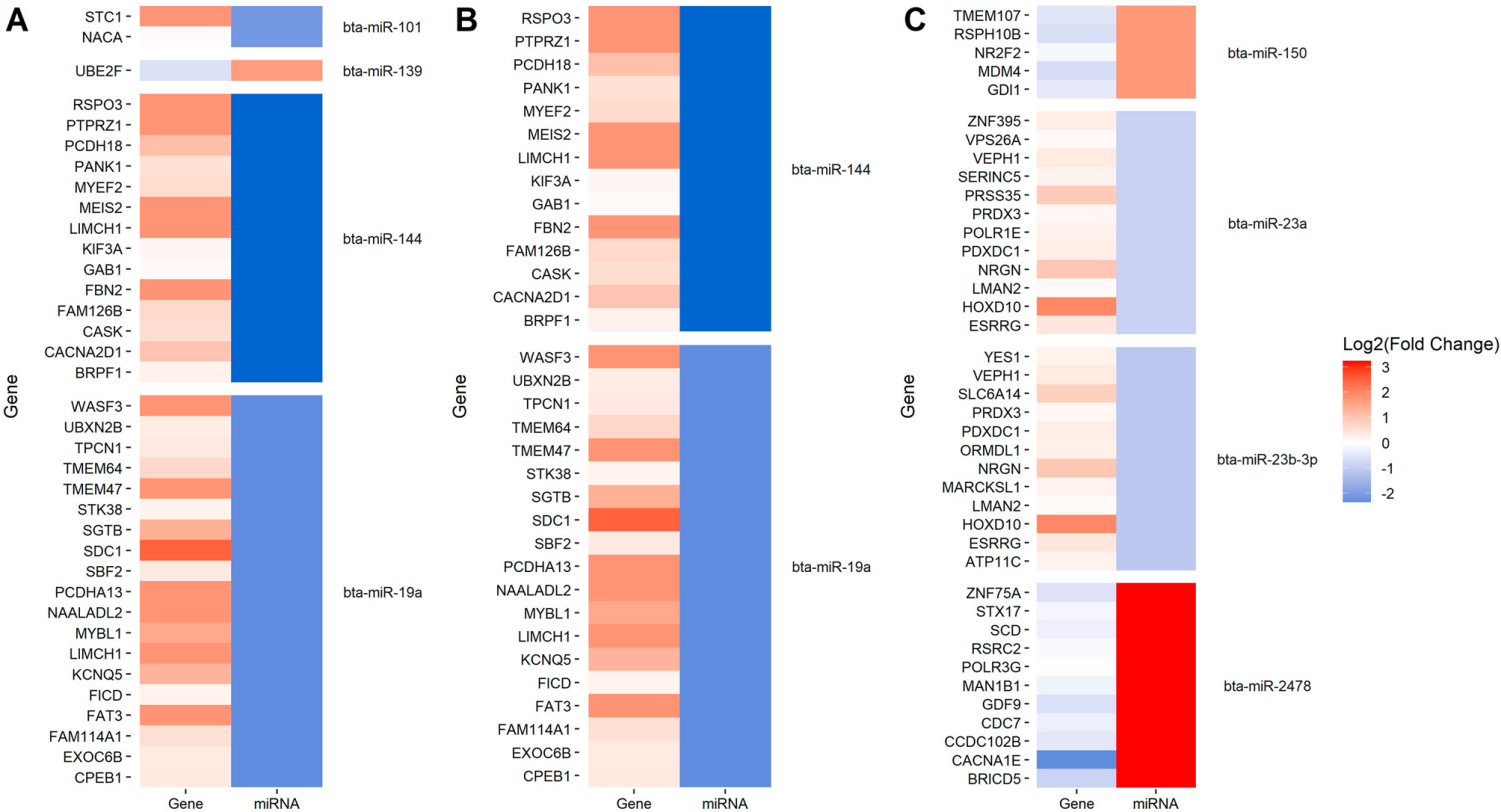
Negative correlations between miRNAs and predicted target mRNAs expression levels. Heatmaps representing changes in the expression of the differentially expressed miRNAs in peripheral blood samples in the comparisons: diffuse lesions vs controls (**A**) and diffuse vs focal lesions (**B**) along with the changes in expression of their predicted target mRNAs. (**C**) Heatmap representing changes in the expression of the differentially expressed miRNAs in ICV samples in the comparison focal vs controls along with the changes in expression of their predicted target mRNAs.

**Figure 5:**
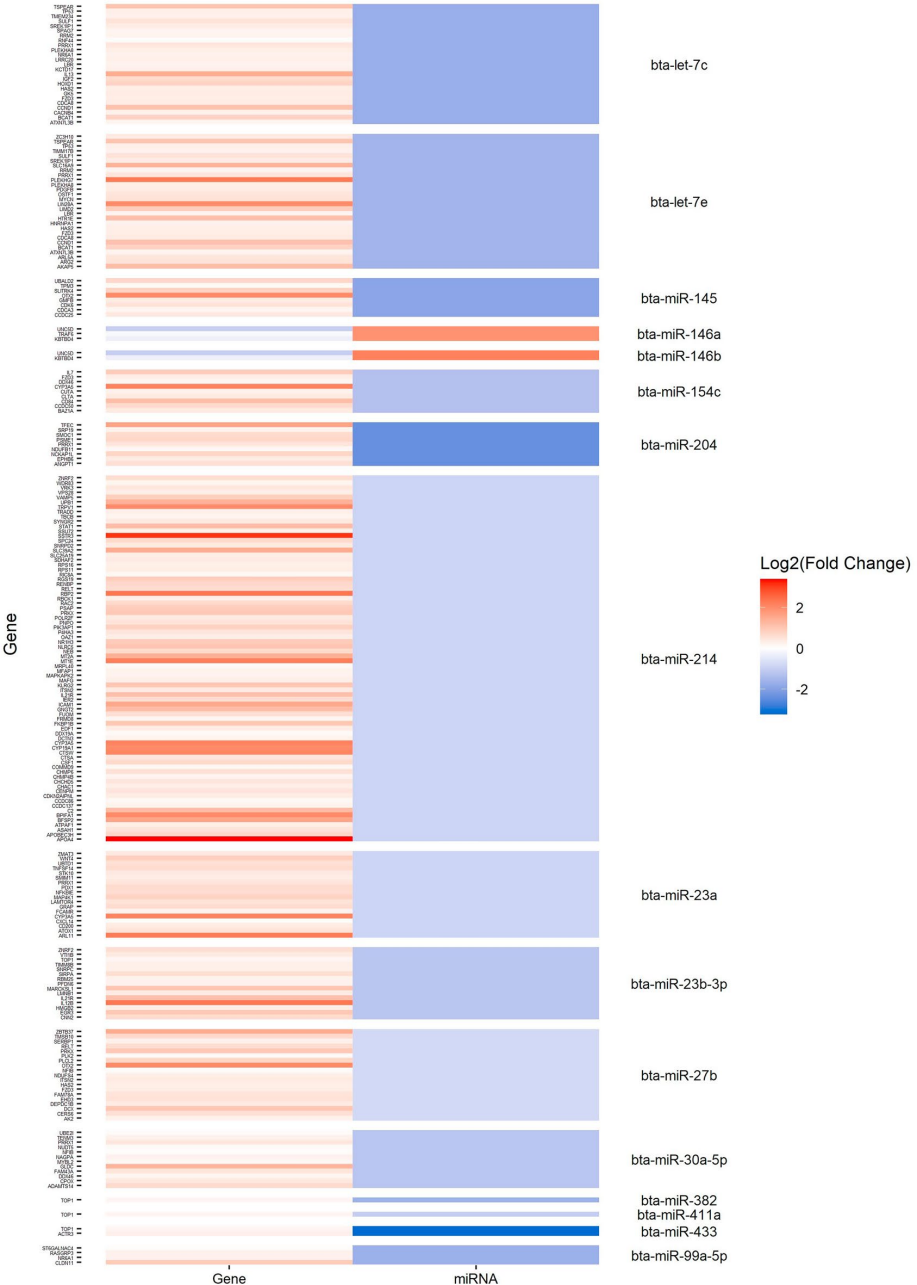
Negative correlations between miRNAs and predicted target mRNAs expression levels. Heatmap representing changes in the expression of the differentially expressed miRNAs in ICV samples in the comparison diffuse vs focal lesions along with the changes in expression of their predicted target mRNAs.

The heatmaps representing the changes in the expression of the differentially expressed miRNAs in the different comparisons along with the changes in the expression of their target mRNAs are presenting in Figs. 4 and 5. As seen in Fig. 4A and B, 32 mRNA targets identified in the two comparisons of PB samples (diffuse lesions vs controls and diffuse vs focal lesions) were upregulated and potentially controlled by only two downregulated miRNAs, bta-miR-144 and bta-miR-19a. In the ICV samples, 33 mRNA targets would be controlled by two upregulated (bta-miR-150 and bta-miR-2478) and two downregulated (bta-miR-23a and bta-miR-23b-3p) miRNAs in the comparison focal lesions vs controls (Fig. 4C). In the comparison of cows with diffuse lesion vs controls, the upregulation of bta-miR-215 (fold = 2.60) would result in the downregulation of the mRNAs *encoding the G Protein-Coupled Receptor 22* (*GPR22*)* and the BMX Non-Receptor Tyrosine Kinase* (*BMX*). Finally, 198 mRNAs were found dysregulated and would be controlled by 16 DE miRNAs in the comparison of cows with diffuse vs focal lesions (Fig. 5).

### Functional enrichment analysis

Functional analysis of all the predicted gene targets was performed by enrichment analysis of GO processes and KEGG pathways (Table 5)*.* Using the predicted gene targets identified in the PB samples, no GOs or pathways could be identified. Using *ClusterProfiler*, two KEGG pathways, the RNA polymerase (bta:03020) and the mitogen-activated protein kinase (MAPK) signaling pathway (bta:04010) were found enriched in the ICV samples of cows with focal lesions vs controls and with diffuse vs focal lesions, respectively. In the comparison of focal lesions vs controls, the *RNA Polymerase I Subunit E* (*POLR1E*) was positively associated with the bta-miR-23a and the *RNA Polymerase III Subunit G* (*POLR3G*) was negatively associated with the bta-miR-2478, the most highly upregulated miRNA in the tested ICV samples (fold = 3.23). In the comparison of diffuse vs focal lesions, downregulation of bta-miR-214, bta-miR-23a, bta-miR-99a-5p, bta-let-7c, bta-miR-204, bta-let-7e caused upregulation of 11 genes belonging to the MAPK signaling pathway (bta04010). In this comparison, the regulation of immune system processes (GO:0002682) and the nucleolus cellular component (GO:0002682) were significantly enriched with 29 and 22 enriched genes in each pathway, respectively. In addition, several KEGG pathways related to the induction of cytokines and inflammatory immune response (WP997) were enriched including the MAPK signaling pathway, pathways in cancer, Kaposi sarcoma-associated herpesvirus infection, Hepatitis C, Measles, Epstein-Barr virus infection, pancreatic cancer, P53 signaling pathway, lysosome, PI3K-Akt, and JAK-STAT signaling pathways.

**Table 5:** Gene ontologies (GOs) and canonical pathways associated with the predicted gene targets for the differentially expressed miRNAs in each comparison.

Comparison	Analysis	GO/pathway	Description	Gene ratio	*p*-adjusted value	Gene ID
Focal lesions vs controls	*Cluster* *profiler*	bta03020	RNA polymerase	2/15	0.03291	*POLR1E, POLR3G*
Diffuse vs focal lesions	*Cluster* *profiler*	bta04010	MAPK signaling pathway	12/105	0.02923	*TRADD, MAPKAPK2, * *MAP4K1, RASGRP3, TP53, TRAF6, ANGPT1, CSF1, RAC2, * *CACNB4, * *IGF2, PDGFB*
	*String*	GO:0002682	Regulation of immune system process	29/1158	0.0094	*CACNB4, NLRC5, CXCL14, * *SIRPA, PLCL2, RAC2, * *CYP19A1, IL13, TRAF6, IL7, * *RBCK1, PIK3AP1, CD200, * *CNN2, EPHB6, TNFSF14, C2, NCKAP1L, CD84, ARG2, * *CSF1, EGR3, IL12B, NR1H3, * *STK10, IGF2, STAT1, CDK6, * *MAPKAPK2*
		GO:0005730	Nucleolus	22/832	0.0138	*ZNRF2, TP53, LIN28A, * *POLR2F, VRK3, SPC24, TOP1, NFIB, CDKN2AIPNL, ZMAT3, SRP19, CCDC86, CDCA8, * *SDHAF2, DCTN3, MRPL40, * *MYCN, CCDC137, NFKBIE, * *EDF1, STAT1, DDX46*
		bta04010	MAPK signaling pathway	12/267	0.0027	*TP53, CACNB4, RAC2, * *TRADD, ANGPT1, TRAF6, * *PDGFB, CSF1, RASGRP3, * *IGF2, MAPKAPK2, MAP4K1*
		bta05200	Pathways in cancer	16/488	0.0027	*TP53, TPM3, RAC2, IL13, * *TRAF6, IL7, CCND1, GNGT2, * *PDGFB, IL12B, RASGRP3, * *IGF2, STAT1, CDK6, FZD3, * *WNT4*
		bta05167	Kaposi sarcoma-associated herpesvirus infection	9/175	0.0046	*TP53, ICAM1, TRADD, * *CCND1, GNGT2, PDGFB, * *STAT1, CDK6, MAPKAPK2*
		bta05160	Hepatitis C	8/150	0.0072	*TP53, TRADD, TRAF6, * *CCND1, CLDN11, NR1H3, * *STAT1, CDK6*
		bta05162	Measles	7/136	0.0197	*TP53, TRADD, TRAF6, * *CCND1, IL12B, STAT1, CDK6*
		bta05169	Epstein–Barr virus infection	8/197	0.0283	*TP53, ICAM1, TRADD, TRAF6, CCND1, NFKBIE, STAT1, * *CDK6*
		bta05212	Pancreatic cancer	5/69	0.0283	*TP53, RAC2, CCND1, STAT1, * *CDK6*
		bta04115	p53 signaling pathway	5/74	0.0289	*TP53, RRM2, ZMAT3, CCND1, CDK6*
		bta04142	Lysosome	6/119	0.0308	*CLTA, NAGPA, ASAH1, CTSA, * *PSAP, CTSW*
		bta04151	PI3K-Akt signaling pathway	10/332	0.0308	*TP53, ANGPT1, IL7, CCND1, * *PIK3AP1, GNGT2, PDGFB, * *CSF1, IGF2, CDK6*
		bta04630	JAK-STAT signaling pathway	7/163	0.0308	*IL13, IL7, CCND1, IL21R, * *PDGFB, IL12B, STAT1*

## Discussion

Previous studies analyzed the differential expression of miRNAs in Holstein cattle experimentally infected with MAP^33,34^. A comparison of the serum miRNome of MAP-challenged IFNɣ responders to unchallenged controls six months after infection did not identify significant differences in miRNA expression^33^. Similarly, Shaughnessy et al. did not detect differentially expressed miRNAs at either the early 6 months or late (43, 46, and 49 months) intervals across seropositive and seronegative animals^34^. Using naturally infected cattle previous studies compared the miRNA profiles of cattle positive and negative for MAP antibodies by ELISA^32^ or compared miRNA profiles according to the stage of MAP infection defined by fecal shedding, ELISA, and clinical signs^53^. In cattle, however, signs of infection such as the appearance of focal lesions in gut tissues can be detected before fecal shedding and ELISA, and therefore, examining the transcriptome of animals according to the histopathological lesions facilitates the identification of animals in the subclinical stage of the infection when the amount of MAP and antibodies can be undetected with current pre-mortem diagnostic methods. In the present study, the absence or presence of PTB-associated lesions (focal or diffuse) was used to define the severity of MAP infection and to compare the miRNA profiles of animals in early and more advanced stages of the infection vs uninfected cattle without lesions in gut tissues. To our knowledge only one study compared miRNA expression in the serum of cattle classified according to histopathological data, serology, and MAP bacterial culture into four disease groups: control, mild, moderate, and severe^54^. Instead of using RNA-Seq, Gupta et al. used NanoString technology (Seattle, WA, US) to detect bovine miRNAs using probe sets specific for human miRNAs. Careful should be taken in generalizing human results to cattle as the miRNome and targets of miRNAs can be organism specific. Indeed, none of the miRNAs detected by Gupta et al., was detected in the current study.

Most previous studies analyzed circulating miRNA profiles in serum samples. In our study, we examined whether the PB miRome recapitulated the miRome of the ICV, the primary site of MAP colonization. None of the identified miRNAs was differentially expressed in both the PB and ICV samples and, therefore, our results showed PB and ICV-specific miRNA expression. Some miRNAs identified in or study matched with the ones identified in previous studies describing miRNA responses to mycobacterial infections including the bta-miR-27a-5p, bta-miR-32, bta-miR-23a, bta-miR-135b, bta-miR146a, bta-miR146b, bta-miR-433, bta-let-7e, and bta-let-7c^32,35,55,56^. Using the predicted gene targets of the miRNAs identified in the PB samples, no GOs or pathways could be identified. However, by analyzing the predicted gene targets of the miRNAs identified in the ICV samples, we were able to distinguish pathways associated with different stages of MAP infection. To our knowledge, our study is the first to analyze miRNAs profiles in tissue samples of MAP-infected cattle.

Our analysis showed that specific miRNAs were dysregulated in cows with different PTB-associated lesions. Specifically, in PB samples, we identified eight miRNAs in the comparison of cows diffuse lesions vs controls and three in the comparison of cows with diffuse vs focal lesions. Importantly, the miRNA-Seq analysis's results and the RT-qPCR results correlated. Among the eight miRNAs differentially expressed in PB samples from cows with diffuse lesions vs controls, three (bta-miR-19a, bta-miR-144, bta-miR-32) were also found downregulated in PB samples of cows with diffuse vs focal lesions. In agreement with these findings, the bta-miR-32 was also found downregulated in a previous study where the miRNA profiles of Holstein cattle positive and negative for MAP antibodies were compared^32^.

In the ICV samples, we identified a total of 4, 5, and 18 miRNAs differentially expressed in cows with focal lesions vs controls, diffuse lesions vs controls, and diffuse vs focal lesions, respectively. This suggests that the miRNA expression in ICV changes more as PTB-associated lesions become more severe. In the comparison of cows with focal lesions vs controls, *DESeq2* was able to identify two upregulated (bta-miR-2478 and bta-miR-150) and two downregulated (bta-miR-23a and bta-iR-23b-3p) miRNAs. Some of the differentially expressed miRNAs in the comparison diffuse lesions vs controls (bta-miR-433, bta-miR-146a, bta-miR-99a-5p) were also dysregulated in the comparison diffuse vs focal lesions. The bta-miR-146a with a conserved human ortholog acts as an inhibitor of the pro-inflammatory immune response^57^. In our study, upregulation of miR-146a would negatively regulate the TNF Receptor Associated Factor 6 (TRAF6) and control the pro-inflammatory immune response^58^. Two miRNAs (bta-miR-23a and bta-miR-23b-3p) were downregulated in both comparisons, focal lesions vs controls and diffuse vs focal lesions.

We also integrated miRNA and mRNA expression data to ascertain whether the differences in miRNA profiles would be reflected in the mRNA data set. The 32 common mRNA targets identified in the two comparisons of PB samples were upregulated in all the comparisons and would be controlled by only two downregulated miRNAs, bta-miR-144 and bta-miR-19a. In ICV samples, 33, 2, and 198 mRNA potential targets were identified in the comparisons focal lesions vs controls, diffuse lesions vs controls, and diffuse vs focal lesions, respectively. Functional analysis using the predicted gene targets allowed the identification of two enriched KEGG pathways, the RNA polymerase (bta:03020) and the mitogen-activated protein kinase (MAPK) signaling pathway (bta:04010), in the ICV samples of cows with focal lesions vs controls and with diffuse vs focal lesions, respectively.

**Figure 6:**
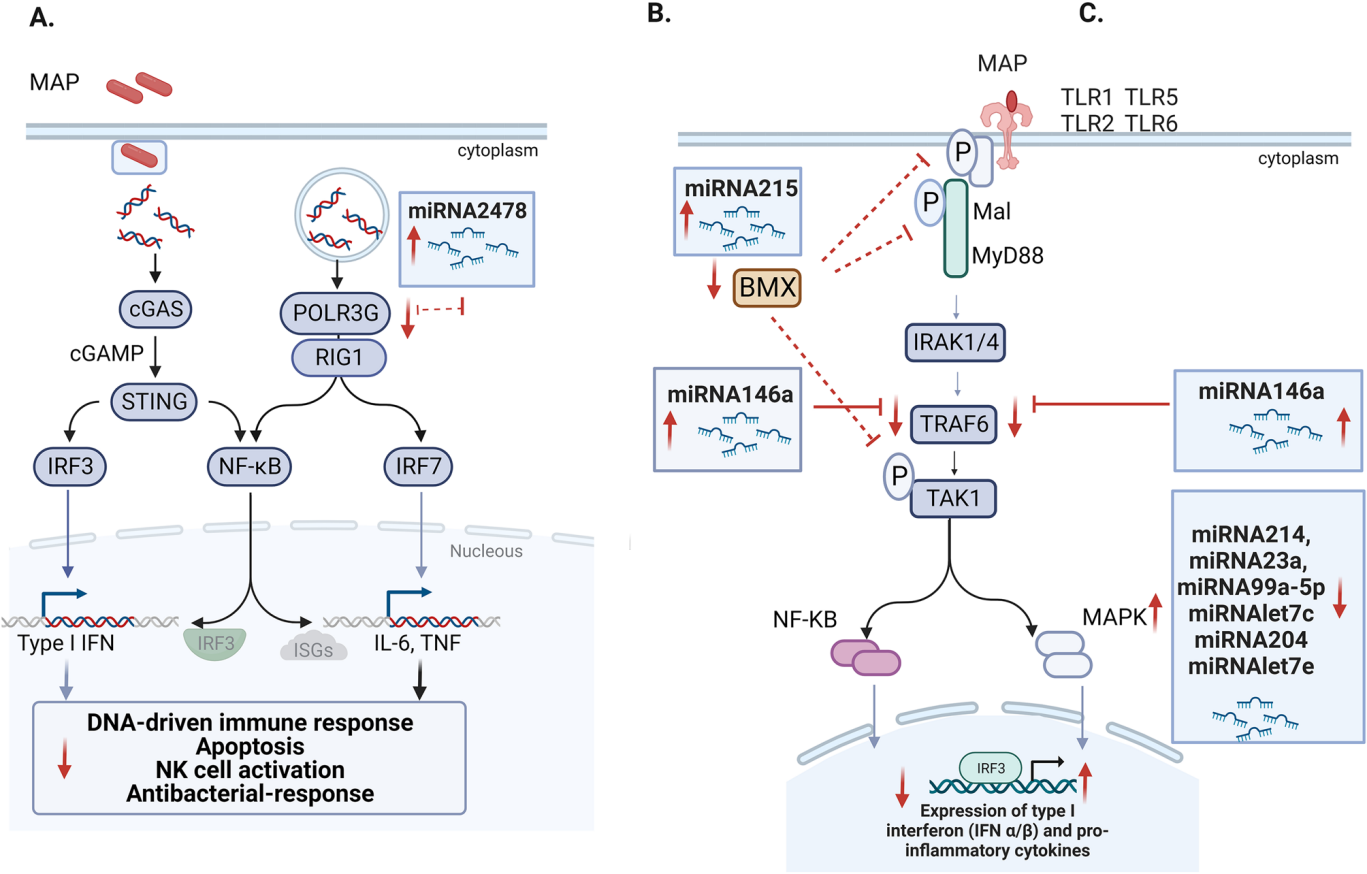
Potential role of the identified miRNAs in the regulation of innate and inflammatory responses in response to MAP infection. (**A**) In the comparison of cows with focal lesions vs controls, the POLR3G would be negatively regulated by the bta-miR-2478. POLR3G is a member of the RNA polymerase III that functions as a non-self dsDNA sensor. The POLR3G downregulation might be responsible for the blockade of the innate immune responses that allow MAP persistence within infected macrophages. (**B**) In the comparison of cows with diffuse vs controls, the upregulation of bta-miR-215 would reduce the expression of BMX and the activation of MAPK and NF-кβ. Upregulation of bta-miR-146a would negatively regulate TRAF6 and the pro-inflammatory response during the subclinical stage of MAP infection. (**C**) In the comparison of cows with diffuse vs focal lesions, however, we observed that the dysregulation of bta-miR-214, bta-miR-23a, bta-miR-99a-5p, bta-let-7c, bta-miR-204, bta-let-7e would upregulate the expression of several genes of the MAPK signaling pathway causing the activation of a strong pro-inflammatory response commonly observed in the clinical stages of the infection. BMX Non-Receptor Tyrosine Kinase (BMX), transforming grow factor-β activated kinase (TAK1), mitogen-activated protein kinase (MAPK), TNF Receptor Associated Factor 6 (TRAF6), interleukin 1 receptor-associated kinase (IRAK), Toll-like receptors (TLR), cyclic GMP-ANP Synthase (cGAS), stimulator of interferon response CGAMP interactor 1 (STING), RNA Polymerase III Subunit G (POLR3G), Interferon regulatory factor 3 (IRF3), nuclear factor (NF-кβ), RNA sensor RIG1, interferon (IFN), tumor necrosis factor (TNF), Interleukin 1 Receptor Associated Kinase 4 (IRAK1/4). Created with BioRender.com.

A more thorough examination of the gene targets in each enriched pathway allowed us to obtain a better understating of the regulation of the innate and inflammatory responses by miRNAs (Fig. 6). In the comparison of cows with focal lesions vs controls (Fig. 6A), the *RNA Polymerase I Subunit E* (*POLR1E*) would be positively regulated by bta-miR-23a, and the *RNA Polymerase III Subunit G* (*POLR3G*) negatively regulated by the bta-miR-2478, the most highly upregulated miRNA in the tested ICV samples (fold = 3.23). POLR3G is part of the RNA polymerase III. POLR3G is involved either in the recruitment and stabilization of the subcomplex within RNA polymerase III, or in stimulating catalytic functions of other subunits during initiation. The RNA polymerase III acts as a nuclear and cytosolic non-self dsDNA sensor, is involved in the positive regulation of innate immune responses and Type I IFN and Nuclear factor (NF-κβ) activation through the RIG1 pathway and plays a key role in sensing and limiting infection by intracellular bacteria and DNA viruses. The POLR3G downregulation might be responsible for the blockade of the innate immune responses generally observed upon MAP infection. This blockage might allow MAP to persist and establish infection within macrophages during the long-term subclinical phase of the infection. The downregulation of POLR3G might be a way for MAP to prolong its survival within infected macrophages by decreasing DNA-driven innate immune responses.

In the comparison of cows with diffuse lesions vs controls (Fig. 6B), the upregulation of bta-miR-215 (fold = 2.60) would result in the downregulation of the mRNAs encoding the *G Protein-Coupled Receptor 22* (*GPR22*)* and* the *BMX Non-Receptor Tyrosine Kinase* (*BMX*). BMX is expressed in hematopoietic cells of the myeloid lineage, granulocytes and monocytes, and has a significant role in cytokine signaling and inflammation^59^. More specifically, BMX is required for the phosphorylation and activation of the Myeloid Differentiation Primary Response protein (MyD88), Mal, and transforming grow factor-β activated kinase (TAK1), which leads to the activation of the NF-кβ and mitogen-activated protein kinase (MAPK) signaling pathways. Impaired production of BMX might therefore result in a control of type I IFN and pro-inflammatory cytokines production. Interestingly, a recent study showed that the downregulation of miR-27a in MAP-infected macrophages significantly inhibited pro-inflammatory responses in MAP infected macrophages by targeting the expression of TAK1 binding proteins 2 and 3 (TAB2/3), important components of the MAPK signaling pathway^60^. In the comparison of cows with diffuse lesions vs controls, we observed that the upregulation of bta-miR-146a was negatively associated with the expression of TRAF6 which in normal conditions would ensure a check-in of the pro-inflammatory signalling of NF-κβ and MAPK. Mir146a expression is induced both in macrophages and in mice after mycobacterial infections, and further supress the iNOs expression and NO generation via NF-кβ and MAPK signalling^61,62^.

In the comparison of cows with diffuse vs focal lesions (Fig. 6C), we observed that the downregulation of bta-miR-214, bta-miR-23a, bta-miR-99a-5p, bta-let-7c, bta-miR-204, bta-let-7e was associated with the upregulation of 12 genes belonging to the MAPK signaling pathway. The MAPK signaling pathway plays a significant role in the induction of pro-inflammatory responses that occur in MAP-infected cattle in advanced stages of the infection^63^. Among the 12 potential gene targets controlled by the differentially expressed miRNAs identified in the comparison diffuse vs focal lesions we found the *Tumor Necrosis Factor Receptor Type 1-associated DEATH Domain Protein* (*TRADD*), *Rac Family Small GTPase 2* (*RAC2*), and *Colony Stimulating Factor 1* (*CSF1*). TRADD mediates programmed cell death, RAC2 is involved in the generation of reactive oxygen species, and CSF1 promotes the release of pro-inflammatory chemokines, and thereby plays an important role in the stimulation of inflammatory processes. In our study, TRADD, RAC2 and CSF1 expression regulation was associated with the bta-mir-214. Besides the MAPK signaling pathway, the software *String* revealed that the target genes identified in the comparison diffuse vs focal lesions were associated with the regulation of the immune system process (GO:0002682) and with the induction of cytokines and inflammatory immune responses (WP997) in several diseases including cancer (bta05200), Kaposi sarcoma-associated herpesvirus infection (bta05167), Hepatitis C (bta05167), Measles (bta05162), Epstein-Barr virus infection (bta05169), pancreatic cancer (bta05212). Signaling pathways associated with inflammation such as the P53 signaling pathway (bta04115), lysosome (bta04142), PI3K-Akt (bta04151), and JAK-STAT (bta04630) were also found significantly enriched.

## Conclusions

Through modulation of the expression of miRNAs, MAP infection modifies host gene expression and modulates the innate and inflammatory responses in distinct stages of the infection. In early stages of the infection, the bta-miR-247 would downregulate POLR3G, an activator of the RNA polymerase III that functions as a non-self dsDNA sensor and is implicated in the activation of DNA-driven innate immune responses. The POLR3G downregulation might be responsible, at least in part, for the blockade of the innate immune responses that allows MAP persistence within infected macrophages during the long-term subclinical stage of the infection. As the infection progresses, MAP grows within granulomas but the upregulation of the bta-miR-215 and bta-miRNA-146a would control the activation of an excessive pro-inflammatory response by targeting BMX and TRAF6, respectively. In clinical stages of the infection, however, the dysregulation of bta-miR-214, bta-miR-23a, bta-miR-99a-5p, bta-let-7c, bta-miR-204, bta-let-7e would upregulate several genes of the MAPK signaling pathway causing the activation of a strong pro-inflammatory response. Aside from the utility of the miRNAs identified in the current study as biomarkers, modifying or targeting the expression of specific miRNAs might serve as a novel strategy for PTB control.

## Data Availability

RNA-Seq data have been deposited in the NCBI Gene Expression Omnibus (GEO) database under the accession number (GSE137395). The datasets generated during the current study are available from the corresponding author on reasonable request.

## References

[1] Ott SL, Wells SJ, Wagner BA (1999). Herd-level economic losses associated with Johne’s disease on US dairy operations. Prev. Vet. Med..

[2] Rasmussen P, Barkema HW, Mason S, Beaulieu E, Hall DC (2021). Economic losses due to Johne’s disease (paratuberculosis) in dairy cattle. J. Dairy Sci..

[3] Nielsen SS, Toft N (2009). A review of prevalences of paratuberculosis in farmed animals in Europe. Prev. Vet. Med..

[4] Juste RA (2009). Association between *Mycobacterium avium* subsp. *paratuberculosis* DNA in blood and cellular and humoral immune response in inflammatory bowel disease patients and controls. Int. J. Infect. Dis..

[5] Pierce ES (2018). Could *Mycobacterium avium* subspecies *paratuberculosis* cause Crohn’s disease, ulcerative colitis and colorectal cancer?. Infect. Agent Cancer.

[6] Dow CT (2021). Warm, sweetened milk at the twilight of immunity—Alzheimer’s disease—Inflammaging, insulin resistance, *M. paratuberculosis* and immunosenescence. Front. Immunol..

[7] Hines ME (2007). Efficacy of spheroplastic and cell-wall competent vaccines for *Mycobacterium avium* subsp. *paratuberculosis* in experimentally-challenged baby goats. Vet. Microbiol..

[8] Bermudez LE, Petrofsky M, Sommer S, Barletta RG (2010). Peyer’s patch-deficient mice demonstrate that *Mycobacterium avium* subsp. *paratuberculosis* translocates across the mucosal barrier via both M cells and enterocytes but has inefficient dissemination. Infect. Immun..

[9] Rees WD, Lorenzo-Leal AC, Steiner TS, Bach H (2020). *Mycobacterium avium* subspecies *paratuberculosis* infects and replicates within human monocyte-derived dendritic cells. Microorganisms.

[10] Khare S (2012). Systems biology analysis of gene expression during in vivo *Mycobacterium avium*
*paratuberculosis* enteric colonization reveals role for immune tolerance. PLoS One.

[11] Whitlock RH, Buergelt C (1996). Preclinical and clinical manifestations of paratuberculosis (including pathology). Vet. Clin. N. Am. Food Anim. Pract..

[12] Clarke CJ (1997). Paratuberculosis and molecular biology. Vet. J..

[13] Stabel JR, Whitlock RH (2001). An evaluation of a modified interferon-γ assay for the detection of paratuberculosis in dairy herds. Vet. Immunol. Immunopathol..

[14] González J (2005). Histopathological classification of lesions associated with natural paratuberculosis infection in cattle. J. Comp. Pathol..

[15] Sweeney RW (2011). Pathogenesis of paratuberculosis. Vet. Clin. N. Am. Food Anim. Pract..

[16] Koets AP, Eda S, Sreevatsan S (2015). The within host dynamics of *Mycobacterium avium* ssp. *paratuberculosis* infection in cattle: Where time and place matter. Vet. Res..

[17] Whitlock RH, Wells SJ, Sweeney RW, Van Tiem J (2000). ELISA and fecal culture for paratuberculosis (Johne’s disease): Sensitivity and specificity of each method. Vet. Microbiol..

[18] Seth M (2009). Biomarker discovery in subclinical mycobacterial infections of cattle. PLoS One.

[19] van den Esker MH, Koets AP (2019). Application of transcriptomics to enhance early diagnostics of mycobacterial infections, with an emphasis on *Mycobacterium avium* ssp. *paratuberculosis*. Vet. Sci..

[20] Ariel O (2021). Genome-wide association analysis identified both RNA-seq and DNA variants associated to paratuberculosis in Canadian Holstein cattle ‘in vitro’ experimentally infected macrophages. BMC Genomics.

[21] Alonso-Hearn M (2019). RNA-Seq analysis of ileocecal valve and peripheral blood from Holstein cattle infected with *Mycobacterium avium* subsp. *paratuberculosis* revealed dysregulation of the CXCL8/IL8 signaling pathway. Sci. Rep..

[22] Gupta P (2019). Analysis of long non-coding RNA and mRNA expression in bovine macrophages brings up novel aspects of *Mycobacterium avium* subspecies *paratuberculosis* infections. Sci. Rep..

[23] Marete A, Ariel O, Ibeagha-Awemu E, Bissonnette N (2021). Identification of long non-coding RNA isolated from naturally infected macrophages and associated with bovine Johne’s disease in Canadian Holstein using a combination of neural networks and logistic regression. Front. Vet. Sci..

[24] Bao Y (2022). Analysis of long non-coding RNA expression profile of bovine monocyte-macrophage infected by *Mycobacterium avium* subsp. *paratuberculosis*. BMC Genomics.

[25] Triantaphyllopoulos KA (2023). Long non-coding RNAs and their “discrete” contribution to IBD and Johne’s disease—What stands out in the current picture? A comprehensive review. Int. J. Mol. Sci..

[26] Duttagupta R, Jiang R, Gollub J, Getts RC, Jones KW (2011). Impact of cellular miRNAs on circulating miRNA biomarker signatures. PLoS One.

[27] Fang Z, Rajewsky N (2011). The impact of miRNA target sites in coding sequences and in 3′UTRs. PLoS One.

[28] Turchinovich A, Weiz L, Burwinkel B (2012). Extracellular miRNAs: The mystery of their origin and function. Trends Biochem. Sci..

[29] Coutinho LL (2007). Discovery and profiling of bovine microRNAs from immune-related and embryonic tissues. Physiol. Genomics.

[30] Gu Z, Eleswarapu S, Jiang H (2007). Identification and characterization of microRNAs from the bovine adipose tissue and mammary gland. FEBS Lett..

[31] Flicek P (2014). Ensembl 2014. Nucleic Acids Res..

[32] Malvisi M (2016). Responses of bovine innate immunity to *Mycobacterium avium* subsp *paratuberculosis* infection revealed by changes in gene expression and levels of microRNA. PLoS One.

[33] Farrell D (2015). The identification of circulating MiRNA in bovine serum and their potential as novel biomarkers of early *Mycobacterium avium* subsp *paratuberculosis* infection. PLoS One.

[34] Shaughnessy RG, Farrell D, Riepema K, Bakker D, Gordon SV (2015). Analysis of biobanked serum from a *Mycobacterium avium* subsp *paratuberculosis* bovine infection model confirms the remarkable stability of circulating mirna profiles and defines a bovine serum mirna repertoire. PLoS One.

[35] Liang G (2016). Altered microRNA expression and pre-mRNA splicing events reveal new mechanisms associated with early stage *Mycobacterium avium* subspecies *paratuberculosis* infection. Sci. Rep..

[36] Blanco-Vázquez C (2020). Detection of latent forms of *Mycobacterium avium* subsp. *paratuberculosis* infection using host biomarker-based ELISAs greatly improves paratuberculosis diagnostic sensitivity. PLoS One.

[37] Andrews, S. *fastQC*. https://www.bioinformatics.babraham.ac.uk/projects/fastqc/.

[38] Krueger, F. *Trim Galore*. https://www.bioinformatics.babraham.ac.uk/projects/trim_galore/.

[39] Dobin A (2013). STAR: Ultrafast universal RNA-seq aligner. Bioinformatics.

[40] Liao Y, Smyth GK, Shi W (2019). The R package Rsubread is easier, faster, cheaper and better for alignment and quantification of RNA sequencing reads. Nucleic Acids Res..

[41] Love MI, Huber W, Anders S (2014). Moderated estimation of fold change and dispersion for RNA-seq data with DESeq2. Genome Biol..

[42] Aparicio-Puerta E (2019). SRNAbench and sRNAtoolbox 2019: Intuitive fast small RNA profiling and differential expression. Nucleic Acids Res..

[43] Rueda A (2015). SRNAtoolbox: An integrated collection of small RNA research tools. Nucleic Acids Res..

[44] Benjamini Y, Hochberg Y (1995). Controlling the false discovery rate: A practical and powerful approach to multiple testing. J. R. Stat. Soc. Ser. B (Methodological).

[45] R Core Team. *R: A Language and Environment for Statistical Computing*. Preprint https://www.r-project.org/ (2023).

[46] Enright AJ (2003). MicroRNA targets in Drosophila. Genome Biol..

[47] Kertesz M, Iovino N, Unnerstall U, Gaul U, Segal E (2007). The role of site accessibility in microRNA target recognition. Nat. Genet..

[48] Agarwal V, Bell GW, Nam JW, Bartel DP (2015). Predicting effective microRNA target sites in mammalian mRNAs. Elife.

[49] Friedman RC, Farh KKH, Burge CB, Bartel DP (2009). Most mammalian mRNAs are conserved targets of microRNAs. Genome Res..

[50] Wu T (2021). clusterProfiler 4.0: A universal enrichment tool for interpreting omics data. Innovation.

[51] Szklarczyk D (2023). The STRING database in 2023: Protein–protein association networks and functional enrichment analyses for any sequenced genome of interest. Nucleic Acids Res..

[52] Brown RAM (2018). Total RNA extraction from tissues for microRNA and target gene expression analysis: Not all kits are created equal. BMC Biotechnol..

[53] Choi SW (2021). MicroRNA profiling in bovine serum according to the stage of *Mycobacterium avium* subsp. *paratuberculosis* infection. PLoS One.

[54] Gupta SK (2018). Detection of microRNA in cattle serum and their potential use to diagnose severity of Johne’s disease. J. Dairy Sci..

[55] Vegh P (2015). MicroRNA profiling of the bovine alveolar macrophage response to *Mycobacterium **bovis* infection suggests pathogen survival is enhanced by microRNA regulation of endocytosis and lysosome trafficking. Tuberculosis.

[56] Furci L, Schena E, Miotto P, Cirillo DM (2013). Alteration of human macrophages microRNA expression profile upon infection with *Mycobacterium tuberculosis*. Int. J. Mycobacteriol..

[57] Chandan K, Gupta M, Sarwat M (2020). Role of host and pathogen-derived microRNAs in immune regulation during infectious and inflammatory diseases. Front. Immunol..

[58] Quinn EM, Wang JH, O’Callaghan G, Redmond HP (2013). MicroRNA-146a is upregulated by and negatively regulates TLR2 signaling. PLoS One.

[59] Chen X-L, Qiu L, Wang F, Liu S (2014). Current understanding of tyrosine kinase BMX in inflammation and its inhibitors. Burns Trauma.

[60] Hussain T (2018). MicroRNA 27a-3p regulates antimicrobial responses of murine macrophages infected by *Mycobacterium avium* subspecies *paratuberculosis* by targeting interleukin-10 and TGF-β-activated protein kinase 1 binding protein 2. Front. Immunol..

[61] Li M (2016). MicroRNA-146a promotes mycobacterial survival in macrophages through suppressing nitric oxide production. Sci. Rep..

[62] Tahamtan A, Teymoori-Rad M, Nakstad B, Salimi V (2018). Anti-inflammatory microRNAs and their potential for inflammatory diseases treatment. Front. Immunol..

[63] Souza CD, Evanson OA, Weiss DJ (2007). Role of the mitogen-activated protein kinase pathway in the differential response of bovine monocytes to *Mycobacterium avium* subsp. *paratuberculosis* and *Mycobacterium avium* subsp. *avium*. Microbes Infect..

